# Major adverse cardiovascular events in older emergency department patients presenting with non-cardiac medical complaints

**DOI:** 10.1007/s12471-022-01700-z

**Published:** 2022-06-07

**Authors:** N. Zelis, A. M. M. Roumans-van Oijen, J. Buijs, D. J. W. van Kraaij, S. M. J. van Kuijk, P. W. de Leeuw, P. M. Stassen

**Affiliations:** 1 Department of Internal Medicine and Gastroenterology, Zuyderland Medical Centre, Sittard-Geleen, The Netherlands; 2 Department of Internal Medicine, Division of General Internal Medicine, Section Acute Medicine, Maastricht University Medical Centre, Maastricht University, Maastricht, The Netherlands; 3 CARIM School for Cardiovascular Diseases, Maastricht University Medical Centre, Maastricht University, Maastricht, The Netherlands; 4 Department of Cardiology, Zuyderland Medical Centre, Sittard-Geleen, The Netherlands; 5 Department of Clinical Epidemiology and Medical Technology Assessment, Maastricht University Medical Center, Maastricht University, Maastricht, The Netherlands; 6 School of Care and Public Health Research Institute, Maastricht University Medical Centre, Maastricht University, Maastricht, The Netherlands

**Keywords:** Older patients, Emergency department, Major adverse cardiovascular events, Biomarkers

## Abstract

**Objective:**

The risk of major adverse cardiovascular events (MACE) for older emergency department (ED) patients presenting with non-cardiac medical complaints is unknown. To apply preventive measures timely, early identification of high-risk patients is incredibly important. We aimed at investigating the incidence of MACE within one year after their ED visit and the predictive value of high-sensitivity cardiac troponin T (hs-cTnT) and N‑terminal pro-B-type natriuretic peptide (NT-proBNP) for subsequent MACE.

**Methods:**

This is a substudy of a Dutch prospective cohort study (RISE UP study) in older (≥ 65 years) medical ED patients who presented with non-cardiac complaints. Biomarkers were measured upon ED arrival. Cox-regression analysis was used to determine the predictive value of the biomarkers, when corrected for other possible predictors of MACE, and area under the curves (AUCs) were calculated.

**Results:**

Of 431 patients with a median age of 79 years, 86 (20.0%) developed MACE within 1 year. Both hs-cTnT and NT-proBNP were predictive of MACE with an AUC of 0.74 (95% CI 0.68–0.80) for both, and a hazard ratio (HR) of 2.00 (95% CI 1.68–2.39) and 1.82 (95% CI 1.57–2.11) respectively. Multivariate analysis correcting for other possible predictors of MACE revealed NT-proBNP as an independent predictor of MACE.

**Conclusion:**

Older medical ED patients are at high risk of subsequent MACE within 1 year after their ED visit. While both hs-cTnT and NT-proBNP are predictive, only NT-proBNP is an independent predictor of MACE. It is likely that early identification of those at risk offers a window of opportunity for prevention.

**Supplementary Information:**

The online version of this article (10.1007/s12471-022-01700-z) contains supplementary material, which is available to authorized users.

## What’s new?

- Older emergency department patients presenting with non-cardiac complaints are at high risk of developing MACE (20%) and cardiovascular death (4.9%) within 1 year
- Older emergency department patients with non-cardiac complaints and high risk of MACE can be identified by biomarkers hs-cTnT and NT-proBNP
- NT-proBNP is an independent predictor of MACE in this population
- Early identification of high-risk patients may offer an opportunity for preventive strategies

## Introduction

Older patients are at high risk of adverse outcomes after an emergency department (ED) visit [1, 2]. However, the risk of major adverse cardiovascular events (MACE) for older ED patients, presenting with non-cardiac medical complaints, is unknown. Because preventive measures may improve outcome [3], early identification of patients at risk is highly important.

Besides conventional cardiovascular risk factors, the cardiac biomarkers high-sensitivity cardiac Troponin T (hs-cTnT) and N‑terminal pro-B-type natriuretic peptide (NT-proBNP) have demonstrated to be predictors of MACE [4–8]. The predictive value of hs-cTnT and NT-proBNP for subsequent MACE in older patients, presenting to the internist with non-cardiac complaints, has not yet been studied.

We hypothesised that ED patients who present with non-cardiac complaints are at high risk of developing MACE and that both NT-proBNP and troponin T are predictors of subsequent MACE. In this prospective study, we investigated the incidence of MACE within 1 year after ED visit in older patients who present with non-cardiac medical complaints. In addition, we investigated the discriminatory value of both biomarkers for subsequent MACE and the incidence of 1‑year all-cause and cardiovascular mortality.

## Methods

### Study design and patient selection

This study is part of the RISE UP study, a prospective multicentre observational cohort study, which aimed to identify predictors of adverse outcome in older ED patients [9]. A secondary analysis was performed on data of patients included in one of the two participating hospitals (Zuyderland Medical Centre [MC]), since biomarkers were routinely assessed at this site. Zuyderland MC is a secondary care teaching hospital in the Netherlands with 635 beds and 30,000 ED visits annually. Patients presenting with cardiac complaints are treated at a Heart Emergency Care unit (*Eerste Hart Hulp—EHH*), which is located elsewhere in the hospital. Patients were included if they were aged 65 or over, were assessed by an internist or gastroenterologist at the ED and had provided written informed consent. Patients were excluded if they were already participating in this study or were unable to speak Dutch, German or English. In addition, all medical files were carefully checked for signs of MACE at the time of the ED visit, and patients were excluded when a MACE was already present at the time of their index visit. All patients were followed for 1 year.

This study was approved by the Medical Ethics Committee of Zuyderland MC (NL55867.096.15) and registered on clinicaltrials.gov (NCT02946398). This study was reported according to the Strengthening the Reporting of Observational Studies in Epidemiology (STROBE) guidelines [10].

### Biomarker measurement

During routine blood sampling, one additional venous blood sample was drawn. Plasma was analysed for hs-cTnT and NT-proBNP and results were only available to the investigators and blinded for all health-care providers. If one of these biomarkers was specifically requested, a different blood sample was analysed and results were reported as usual.

### Data collection

Data were retrieved from electronical medical records. We collected the following possible predictors of MACE [3, 11–14]: age, sex, cardiovascular comorbidities, cardiovascular risk factors, presentation with infection, laboratory tests and electrocardiograms (ECGs). The following cardiovascular comorbidities were retrieved: atrial fibrillation, cerebrovascular accident (CVA), transient ischaemic attack (TIA), peripheral vascular disease, myocardial infarction and heart failure according to the Charlson Comorbidity Index (CCI) [15]. In addition, we retrieved data on cardiovascular risk factors: hypertension, statin use (as surrogate marker of hypercholesterolaemia), diabetes mellitus, obesity (body mass index (BMI) > 30 kg/m^2^), current smoking status, and a family history of cardiovascular disease. Data regarding the use of medication were retrieved. Diabetes mellitus was considered present when patients were treated with glucose-lowering drugs. If data regarding cardiovascular risk factors were lacking, these risk factors were assumed to be absent. The definite diagnosis for the ED visit was categorised according to the International Classification of Diseases (ICD)-10 [16]. The following laboratory tests were collected: renal function, C‑reactive protein (CRP), hs-cTnT and NT-proBNP. ECGs made during the ED visit were collected and analysed by an experienced cardiologist using the criteria of the Simplified ECG score [11]. This score is derived by the summation of 12 ECG abnormalities including left and right ventricular hypertrophy, bundle branch block, ST depression, atrial fibrillation, and prolonged QTc interval (Table S1 in Electronic Supplementary Material [ESM]). An ECG was considered abnormal when the simplified ECG score was > 0.

Lastly, data on MACE and mortality occurring during the 1‑year follow-up were collected. MACE was defined as the first manifestation of cardiac arrest, myocardial infarction, arrhythmia requiring intervention (excluding atrial fibrillation, atrial flutter and other arrythmias, which were treated using anti-arrhythmic medication), ischaemic or haemorrhagic stroke, TIA, heart failure, cardiac and peripheral revascularisation procedures or death attributable to a cardiovascular cause. These were derived from medical records, which are connected to the municipal administrational records. We checked all medical files for MACE during follow-up and checked if a MACE was not missed during the time of the ED visit. Most patients returned to the hospital for follow-up by a specialist. In case a patient did not visit the hospital within one year after the ED visit we contacted the general practitioner for information regarding the occurrence of MACE.

Since silent MACE could still have been present during ED presentation in patients who later experienced a MACE, we derived information regarding the results of echocardiograms which were made after the index ED visit during the 1‑year follow-up.

### Outcome measures

The primary endpoint was the incidence of a subsequent MACE within 1 year. Secondary endpoints were 1‑year all-cause mortality and cardiovascular mortality. All patients were followed up for 1 year.

### Data analysis

The sample size available for this study depended on the RISE UP study, which provided data on 450 patients [9]. We performed descriptive analyses of selected patients. To evaluate possible selection bias, we compared baseline characteristics between included and non-included patients.

Area under the curves (AUCs) for receiver operating characteristics for hs-cTnT and NT-proBNP were calculated. Kaplan-Meier survival curves and log-rank tests were used to analyse differences in time to first MACE for both biomarkers, after dividing them into tertiles, and to test for differences in 1‑year all-cause mortality between patients with and without MACE.

Possible predictors of MACE were entered in a univariate Cox regression analysis and hazard ratios (HRs) with 95% confidence intervals (CIs) were calculated. Missing items were imputed using stochastic regression with predictive mean matching (Table S1 in ESM). All variables were tested for the proportional hazards assumption by testing the influence of a time dependent Cox model and for collinearity using the Pearson’s correlation coefficient. Continuous variables were used linearly or log transformed when indicated. In the multivariable analysis, we corrected NT-proBNP and hs-cTnT for other possible predictors of MACE. In addition, we repeated the analysis after removal of patients where a silent MACE could not be ruled out during the ED visit (based on additional data of follow-up echocardiograms). *P*-values below 0.05 were considered statistically significant for all statistical tests. All data were analysed using SPSS Statistics for Windows version 24.0 (IBM Corp., Armonk, N.Y., USA).

## Results

### Study population and patient characteristics

In total, 450 patients were included in the RISE UP study [17] in Zuyderland MC (Fig. S1 in ESM). Nineteen patients were excluded because a MACE was present at the time of ED visit. The remaining 431 patients had a median age of 79 (interquartile range [IQR] 73–85) years and 53.1% was male (Tab. 1). More than half of the patients had at least one cardiovascular comorbidity (52.4%) or at least one cardiovascular risk factor (85.3%). The main reason for ED visit was infectious disease (*n* = 127, 29.5%). In total 177 patients (41.1%) presented with an infection, of which 121 (68.4%) presented with sepsis including 8 (6.6%) with septic shock. Most patients (60.4%) had an abnormal ECG, and hs-cTnT and NT-proBNP values were above reference range in 69.5% and 34.8% of the patients respectively. The most common ECG abnormalities were left-axis deviation, ST depression and atrial fibrillation (Table S1 in ESM). In total, 347 (80.5%) patients were admitted to hospital following the ED visit. Follow-up was complete for all patients. When comparing the baseline characteristics of included and non-included patients, we found no relevant differences in baseline characteristics and outcomes (Table S2 in ESM).

**Table 1:** Baseline characteristics of study participants

Characteristic	Reference values	*N*	Total *n* = 431
*Demographic*			
– Age, median (IQR)		431	79 (73–85)
– Sex, male		431	229 (53.1)
*Cardiovascular comorbidity*			
– Atrial fibrillation		431	123 (28.5)
– CVA/TIA		431	84 (19.5)
– Peripheral vascular disease		431	76 (17.6)
– Myocardial infarction		431	52 (12.1)
– Heart failure		431	40 (9.3)
– At least 1 cardiovascular comorbidity^a^		431	226 (52.4)
*Cardiovascular risk factors*			
– Hypertension		431	220 (51.0)
– Statin use		431	212 (49.2)
– Diabetes mellitus		431	114 (26.5)
– BMI > 30		407	88 (21.6)
– Current smoking		431	54 (12.5)
– Family history of CVD		431	30 (7.0)
– At least one CV risk factor^b^		428	365 (85.3)
*Reason for ED visit (ICD-10)*		431	
– Infectious and parasitic disease			127 (29.5)
– Diseases of the digestive system			124 (28.8)
– Neoplasms			44 (10.2)
– Endocrine and metabolic diseases			25 (5.8)
– Diseases of the circulatory system^c^			24 (5.6)
– Diseases of the respiratory system			23 (5.3)
– Diseases of blood and blood-forming organs			19 (4.4)
– Diseases of the genitourinary system			19 (4.4)
– Miscellaneous			26 (6.0)
*Infection during ED visit*		431	177 (41.1)
*Sepsis during ED visit*		431	121 (28.1)
– Septic shock		121	8 (6.6)^d^
*VTE*		431	18 (4.2)
*ECG*			
– Simplified ECG score, median (IQR)		331	1 (0–2)
– Simplified ECG score > 0		331	200 (60.4)
*Routine laboratory results*			
**–** MDRD, ml/min/1.73 m^2^, median (IQR)	> 90	428	56 (39–75)
**–** MDRD, ml/min/1.73 m^2^, < 60		428	235 (54.9)
– CRP, mg/l, median (IQR)	< 10	427	30 (7–88)
*Biomarkers*			
– hs-cTnT, ng/l, median (IQR)	< 14	406	22 (13–41)
– hs-cTnT above reference range	≥ 14	406	282 (69.5)
– NT-proBNP, ng/l, median (IQR)	< 125	405	786 (288–2521)
– NT-proBNP above age-adjusted limit^e^	Age adjusted	405	141 (34.8)
*Admission*		431	347 (80.5)

Values are* n* (%) unless stated otherwise

*BMI* body mass index, *CVD* cardiovascular disease, *CVA* cerebrovascular accident, *CRP* C-reactive protein, *ECG* electrocardiogram, *ED* emergency department, *hs-cTnT* high-sensitivity cardiac troponin T, *ICD-10* International Classification of Diseases-10, *IQR* interquartile range, *MACE* major adverse cardiovascular events, *MDRD* Modification of Diet in Renal Disease, *NT-proBNP* N-terminal pro-B-type natriuretic peptide, *TIA* transient ischaemic attack, *VTE* venous thromboembolism

^a^Defined as at least ≥ 1 of following cardiovascular comorbidities: atrial fibrillation, CVA/TIA, peripheral vascular disease, myocardial infarction and/or heart failure

^b^Defined as at least ≥ 1 of following cardiovascular risk factors: hypertension, statin use, diabetes mellitus, BMI > 30, current smoking and/or a family history of CVD

^c^Circulatory diseases that are not considered MACE (e.g. venous thromboembolisms)

^d^Denominator count is total number of patients with sepsis (*n* = 121)

^e^For heart failure, limits are age-dependent [24]: 50–75 years:< 900 ng/l; > 75 years < 1800 ng/l

In total, 86 patients (20.0%) developed a MACE within 1 year. The median time to the occurrence of MACE was 50 days (IQR of 6 to 160 days) (Tab. 2). Heart failure (52.3%) and CVA/TIA (26.7%) were the most prevalent first presentations of MACE.

**Table 2:** Outcomes

Outcomes	All patients *n* = 431
*MACE*	86 (20.0)
– Type of MACE^a^	
a. Heart failure	45 (52.3)
b. CVA/TIA	23 (26.7)
c. Myocardial infarction	5 (5.8)
d. Revascularisation	4 (4.7)
e. Cardiovascular death	4 (4.7)
f. Arrhythmia with intervention	3 (3.5)
g. Asystole	2 (2.3)
– Time to MACE, days, median (IQR)	50 (6–160)
*All-cause 1‑year mortality*	139 (32.3)
– Time to death, days, median (IQR)	72 (20–167)
*CV mortality*	21 (15.1)^b^
– Time to death from CV causes, days, median (IQR)	82 (26–293)

Values are *n* (%) unless stated otherwise

*CV* cardiovascular, *CVA* cerebrovascular accident, *MACE* major adverse cardiovascular events, *TIA* transient ischaemic attack

^a^Denominator for the types of MACE is the total number of MACE (*n* = 86)

^b^Denominator for CV mortality is number of all-cause mortality (*n* = 139)

### Discriminatory value of hs-cTnT and NT-proBNP with regard to MACE

**Fig. 1:**
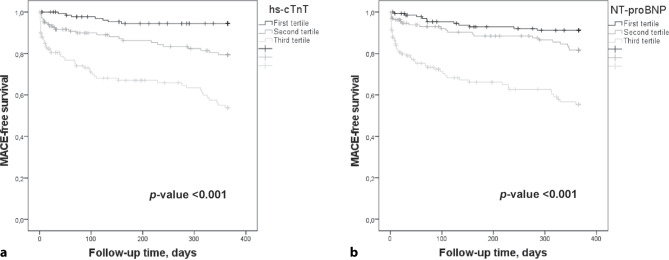
*Kaplan-Meier curves of MACE-free survival. *Kaplan Meier curves of MACE-free survival according to the tertiles of hs-cTnT (**a**) and NT-proBNP (**b**). The tertile values of hs-cTnT were 0–14.5 (first tertile), > 14.5–33.0 (second tertile) and > 33.0 ng/L (third tertile) and of NT-proBNP were 0–388 (first tertile), > 388–1668 (second tertile) and > 1668 ng/L (third tertile)

The AUCs of both hs-cTnT and NT-proBNP for MACE were 0.74 (0.68–0.80). The Kaplan-Meier curves (Fig. 1a and b) showed that most MACEs occurred in patients with biomarker levels in the 3rd tertile (> 33.0 ng/l for hs-cTnT and > 1668 ng/l for NT-proBNP), and least in the 1st tertile (≤ 14.5 ng/l for hs-cTnT and ≤ 388 ng/l for NT-proBNP; *p*-value < 0.001).

### Cox regression analysis

For none of the included variables, the Cox proportional hazard assumption was violated and none were highly correlated. Biomarkers hs-cTnT and NT-proBNP were logarithmically transformed and age, the Simplified ECG score and MDRD were used linearly in the analysis. In the univariable analysis, hs-cTnT and NT-proBNP were both predictive of a subsequent MACE with an HR of 2.00 (95% CI 1.68–2.39) and 1.82 (95% CI 1.57–2.11) respectively (Tab. 3).

**Table 3:** Univariable and multivariable cox regression analysis for MACE

Predictors	Univariable Cox regression		Multivariable Cox regression	
	HR (95% CI)	*p*-value	Corrected HR (95% CI)	*p*-value
Age—per year	1.07 (1.04–1.10)	< 0.001		
Male	1.31 (0.85–2.01)	0.224		
CV comorbidity and risk factors				
– Atrial fibrillation	2.80 (1.83–4.27)	< 0.001		
– CVA/TIA	1.23 (0.74–4.04)	0.430		
– Peripheral vascular disease	1.68 (1.03–2.72)	0.037		
– Myocardial infarction	1.47 (0.83–2.60)	0.189		
– Heart failure	4.63 (2.85–7.54)	< 0.001		
– Hypertension	1.66 (1.07–2.56)	0.023		
– Statin use	0.72 (0.47–1.10)	0.130		
– Diabetes Mellitus	1.72 (1.11–2.67)	0.016		
– BMI > 30	0.79 (0.46–1.34)	0.376		
– Current smoking	1.13 (0.61–2.07)	0.700		
– Family history with CVD	2.00 (1.06–3.77)	0.032		
Presentation with infection	1.56 (1.01–2.41)	0.044		
Simplified ECG score—per point	1.46 (1.27–1.68)	< 0.001		
MDRD—per ml/min/1.73 m^2^	0.97 (0.96–0.98)	< 0.001		
Hs-cTnT—per log ng/l	2.00 (1.68–2.39)	< 0.001	1.05 (0.77–1.44)^a^	0.742
NT-proBNP—per log ng/l	1.82 (1.57–2.11)	< 0.001	1.35 (1.09–1.67)^a^	0.006

*BMI* body mass index, *CI* confidence interval, *CVD* cardiovascular disease, *CVA* cerebrovascular accident, *ECG* electrocardiogram, *hs-cTnT* high-sensitivity cardiac troponin T, *HR* hazard ratio, *MACE* major adverse cardiovascular events, *MDRD* Modification of Diet in Renal Disease, *NT-proBNP* N-terminal pro-B-type natriuretic peptide, *TIA* transient ischaemic attack

^a^Adjusted for covariates: age, sex, atrial fibrillation, CVA/TIA, peripheral vascular disease, myocardial infarction, heart failure, hypertension, statin use, diabetes mellitus, BMI > 30, current smoking, family history of CVD, presentation with infection, Simplified ECG score, MDRD and either NT-proBNP or hs-cTnT

In the multivariable analysis, when corrected for other cardiovascular risk factors, NT-proBNP was independently associated with MACE with an HR of 1.35 (95% CI 1.09–1.67), while hs-cTnT was not (HR of 1.05 (0.77–1.44), Tab. 3).

### 1-year all-cause and cardiovascular mortality

In total, 139 patients (32.3%) died within 1 year of the ED visit (Tab. 2) of whom 21 (of 139; 15.1%) died of cardiovascular causes. The median time from ED presentation until death was 72 days (IQR 20–167) and until death from cardiovascular causes 82 days (IQR 26–293). Patients who developed MACE had a higher 1‑year mortality compared with patients who did not (46.5 vs 28.7% respectively, *p*-value 0.003, Fig. S2).

### Echocardiogram during follow-up and possible silent MACE at time of the index ED visit

In 41 (47.7%) patients with MACE, an echocardiogram was performed during the 1‑year follow-up. The median time to echocardiogram after the ED visit was 76 days (IQR 17–288 days). In the majority (*n* = 28, 68.3%) of these patients, the echocardiogram was abnormal. In only a minority of patients, these finding could be compared with previous echocardiograms. Based on the data of the echocardiograms, a silent MACE at the time of the ED presentation could not be ruled out in 18 patients. When these patients were excluded from the Cox regression analysis, comparable results were found with regard to the predictive value of the biomarkers (HR of 1.03 (95% CI 0.73–1.45) for hs-cTnT and 1.40 (95% CI 1.1–1.77) for NT-proBNP after adjustment for other cardiovascular risk factors).

## Discussion

In this prospective study, we demonstrated that older patients are at high risk (20.0%) of developing MACE within 1 year after visiting the ED for non-cardiac complaints. The median time to MACE was 50 days, with heart failure (54.6%) and CVA/TIA (26.7%) being the most prevalent presentations. Both hs-cTnT and NT-proBNP at the index visit were predictive of subsequent MACE (AUC of 0.74 for both), but only NT-proBNP proved to be an independent predictor of MACE. One-year mortality was high (32.3%), and of these deaths, approximately 15% were certainly due to cardiovascular causes. In patients who developed MACE, 1‑year mortality was higher than in those who did not (46.5 vs 28.7% respectively).

One out of five ED patients developed MACE within 1 year, which appears a very high proportion considering that they presented with non-cardiac complaints. This high incidence may be due to high prevalence of cardiovascular comorbidities and risk factors in our patients and due to our definition of MACE, which also included heart failure without the need of hospital admission. In addition, approximately one third of our patients presented to the ED with an infection. Patients with infections and chronic inflammation are at high risk of developing cardiovascular disease with even higher risks in septic patients [3, 14, 18, 19]. The mechanism of interaction between infection/inflammation and cardiovascular disease is complex, and may be associated with myocardial damage by proinflammatory cytokines or bacterial pathogens, coagulation disorders, induction of hypoxaemia and/or progression of atherosclerosis [20, 21]. Furthermore, MACE during ED presentation could have been missed. We opinion that a combination of cardiovascular risk factors, a chronic inflammatory state due to ageing [19] and/or chronic diseases [22], and an acute medical problem (with or without an acute inflammatory state and possible silent myocardial ischaemia) probably contributed largely to the high subsequent occurrence of MACE in our patients.

To the best of our knowledge, no study examined the predictive ability of hs-cTnT and NT-proBNP for subsequent MACE in older medical ED patients who present with non-cardiac complaints. Apart from several cardiac diseases, hs-cTnT and NT-proBNP can be elevated due to non-cardiac conditions, including sepsis, renal failure and pulmonary embolisms [23, 24], which could consequently influence their predictive value. We found that these biomarkers were predictive of MACE (AUC of 0.74 for both), which is in line with other studies performed in other clinical settings (e.g. in patients with acute cardiac problems, pneumonia or on haemodialysis) [5–7, 25–27]. We further found that NT-proBNP was an independent predictor of MACE, while hs-cTnT was not, which was found in other studies as well [7, 25]. In patients with an acute coronary syndrome [7], NT-proBNP and the GRACE Score were found to be strong independent predictors of MACE 30 days after the ED visit (odds ratio 2.90 for NT-proBNP), while Troponin T was not found to be an independent predictor. In addition, in asymptomatic haemodialysis patients [25], NT-proBNP had a strong relationship with all-cause and cardiovascular mortality, whereas for Troponin T this relationship was weaker. Several factors may explain this independent and compelling predictive power. First, half of the MACEs were due to heart failure and approximately 25% of patients with MACE had a history of heart failure. Secondly, non-cardiac medical conditions which could increase the risk of MACE (e.g. ageing, hyperthyroidism, renal failure, sepsis, shock and pulmonary embolism), may have caused elevated NT-proBNP levels [24, 27], and many of these conditions were present in our patients. Thirdly, since NT-proBNP is released in a reaction to increased ventricle filling pressure, NT-proBNP may be an early marker of a state in which the heart is unable to adjust to a situation with a higher cardiovascular demand (e.g. during an episode of infection, sepsis or hypoxia). We conclude that NT-proBNP is a useful independent predictor of subsequent MACE in older non-cardiac ED patients.

One third of our patients died within 1 year after their ED visit, which definitely reflects the vulnerability of this older population. Only 15% of these deaths were certainly due to cardiovascular causes which is relatively low. However, cardiovascular mortality was probably underestimated since in most patients, no post-mortem examinations were performed. Illustrative of this underestimation may be the finding that patients who developed MACE, more frequently died within 1 year compared with patients who did not. As the incidence of MACE and 1‑year mortality in older ED patients is so high, we think it could be worthwhile to closely monitor high-risk patients after their ED visit in an attempt to improve their outcomes.

### Implication for clinical practice

Since NT-proBNP is an independent predictor of subsequent MACE, it appears likely that patients with elevated NT-proBNP levels may benefit from cardiovascular check-up (e.g. consultation of a cardiologist with possible echocardiography). Therefore, additional prospective research will be necessary to find out if additional cardiovascular check-ups can be helpful in reducing the occurrence of MACE in older medical emergency department patients, presenting with non-cardiac complaints.

### Study strength, limitations and future perspectives

The strength of our study is its prospective design in an older ED population presenting with non-cardiac complaints and the measurement of biomarkers regardless of the reason for ED visit. A limitation of this study is that we cannot rule out that in some patients MACE was missed during the index visit (silent MACE), which could have affected our results. However, we carefully checked all medical files for complaints indicative for MACE during the ED visit and we found similar results after removal of patients where we could not rule out a silent MACE during the ED visit.

Lastly, as physicians had to give priority to providing emergency care, not every possible candidate could be included in the study. We therefore investigated possible selection bias by comparing baseline characteristics and outcomes of the included with non-included patients and found no evidence for this [17].

## Conclusions

Older medical patients who present to the ED with non-cardiac medical complaints are at high risk of developing MACE and dying within 1 year after their ED visit. Both hs-cTnT and NT-proBNP are predictors of subsequent MACE in this ED population, while only NT-proBNP is an independent predictor of MACE. Prospective studies are needed to investigate whether specific or adjusted treatments are able to prevent MACE in these medical patients and if this subsequently improves their outcome.

## Supplementary Information

Fig. S1 Flow chart of patient selection

Fig. S2 Survival curve of patients with and without MACE

Supplemental file with three tables
– Supplemental Table S1 with ECG abnormalities according to the Simplified ECG score
– Supplemental Table S2 with baseline characteristics of the included and non-included patients
– Supplemental Table S3 with results of follow up echocardiogram in patients with MACE
