# Supplementary figures and images for: Major adverse cardiovascular events in older emergency department patients presenting with non-cardiac medical complaints
Source: Neth Heart J. 2022 Jun 7;30(12):559–66. doi: 10.1007/s12471-022-01700-z (PMC9691805; doi:10.1007/s12471-022-01700-z)

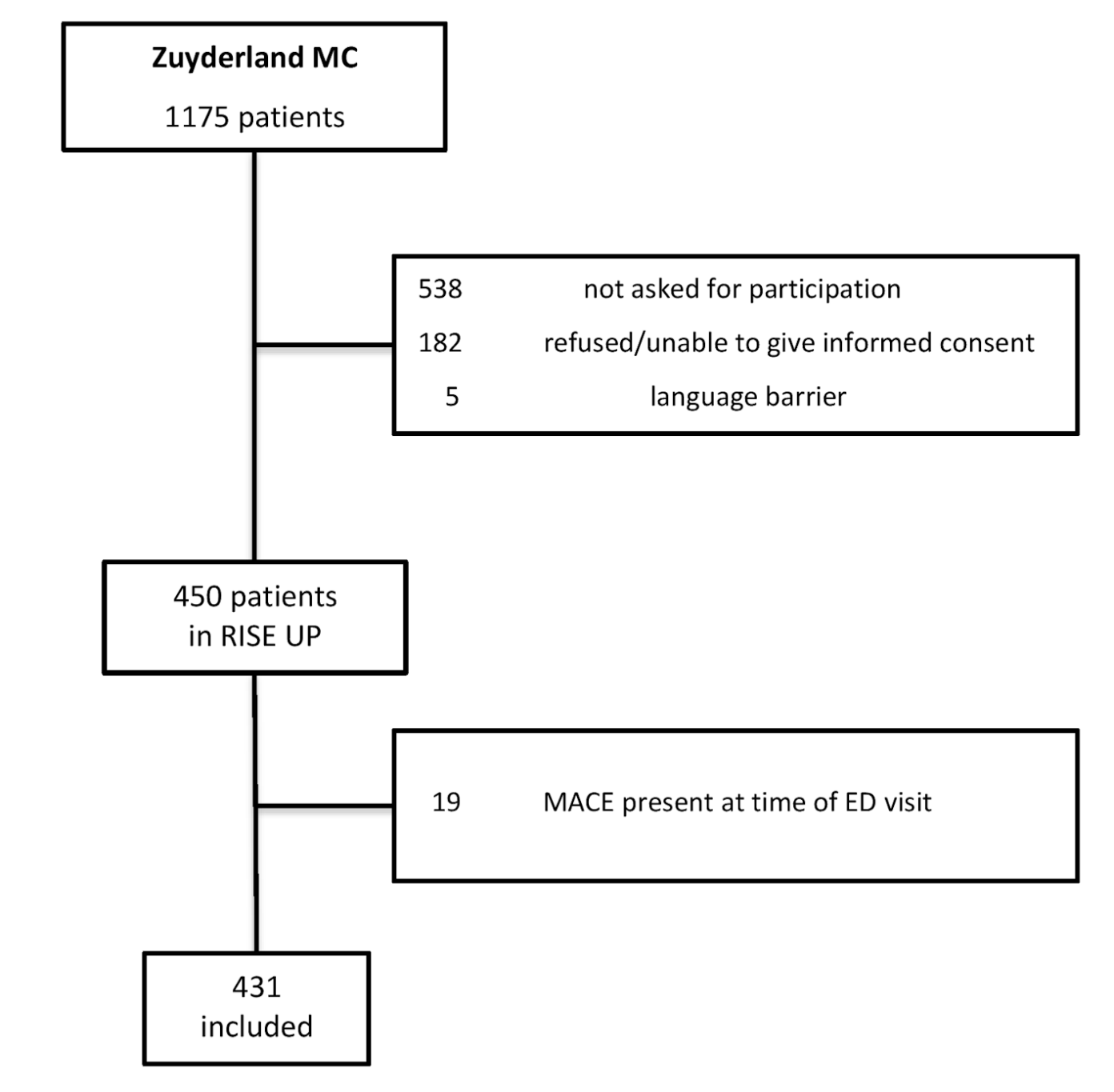

Supplement: Supplementary file 1 — Fig. S1 Flow chart of patient selection [file 12471_2022_1700_MOESM1_ESM.docx]

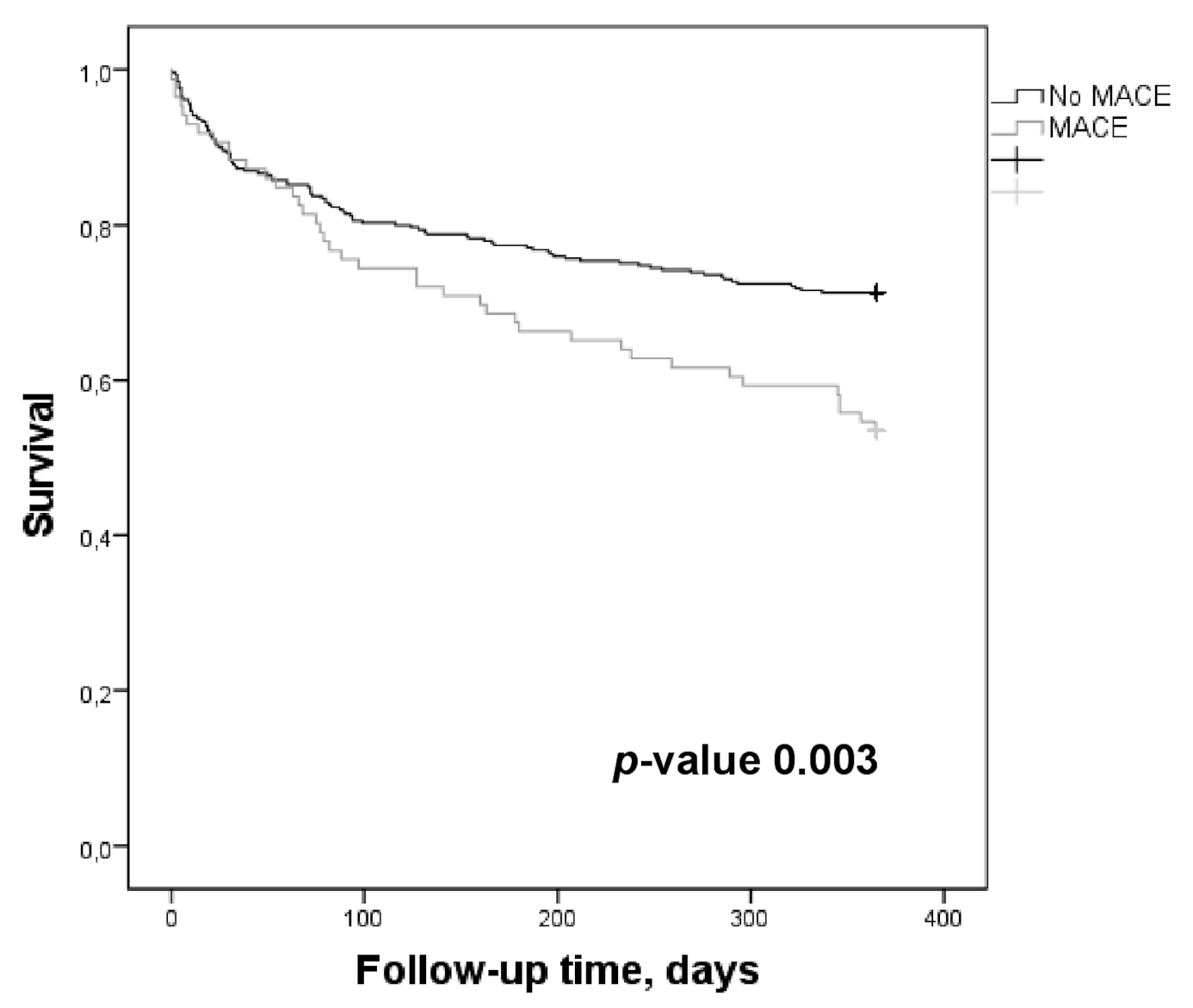

Supplement: Supplementary file 2 — Fig. S2 Survival curve of patients with and without MACE [file 12471_2022_1700_MOESM2_ESM.docx]
