# Supplementary material for: Major adverse cardiovascular events in older emergency department patients presenting with non-cardiac medical complaints
Source: Neth Heart J. 2022 Jun 7;30(12):559–66. doi: 10.1007/s12471-022-01700-z (PMC9691805; doi:10.1007/s12471-022-01700-z)
Supplement: Supplementary file 3 — Supplemental file with three tables – Supplemental Table S1 with ECG abnormalities according to the Simplified ECG score – Supplemental Table S2 with baseline characteristics of the included and non-included patients – Supplemental Table S3 with results of follow up echocardiogram in patients with MACE [file 12471_2022_1700_MOESM3_ESM.docx]

**Supplemental File**

**Major adverse cardiovascular events in older emergency patients with**

**non-cardiac complaints**

N Zelis, MD; AMM Roumans-van Ooijen, MD; J Buijs, MD PhD; DJW van Kraaij, MD PhD; SMJ van Kuijk, PhD; PW de Leeuw, MD PhD; PM Stassen, MD PhD

**Table of contents**

Supplemental Table S1…..………………………………………………………………………………………………p2

Supplemental Table S2…………………………………………………………………………………………………..p3

Supplemental Table S3…………………………………………………………………………………………………..p4

| **Supplemental Table S1** *ECG abnormalities* | |
| --- | --- |
| **ECG abnormality according to the Simplified ECG Score[1]** | ***n*=331** |
| Left axis deviation | 73 |
| ST-depression | 70 |
| Atrial fibrillation | 59 |
| Intraventricular conduction delay | 49 |
| Left ventricle hypertrophy | 43 |
| Prolonged QTc interval | 36 |
| Presence of Q-wave | 26 |
| Right bundle branch block | 23 |
| Left bundle branch block | 22 |
| Right axis deviation | 11 |
| Left atrial abnormality | 8 |
| Right ventricle hypertrophy | 7 |

*ECG* electrocardiogram, *QTc* corrected QT interval

| **Supplemental Table S2** *Baseline characteristics of the included and non-included patients^a^* | | |
| --- | --- | --- |
| **Characteristic** | **Included**  **(*n*= 450)** | **Non-included^b^**  **(*n*=200)** |
| Median age (IQR), years | 79 (73-85) | 78 (72–85) |
| Male sex, n (%) | 234 (52.0) | 100 (50.0) |
| Living in nursing- or care home, n (%) | 39 (8.7) | 23 (11.7) |
| Median CCI score (IQR) | 2 (1-3) | 2 (1-3) |
| Manchester triage category, n (%) |  |  |
| Orange | 56 (12.5) | 38 (19.2) |
| Yellow | 254 (56.8) | 108 (54.5) |
| Green | 133 (29.8) | 51 (25.8) |
| Treatment, n (%) |  |  |
| Internal medicine | 326 (72.4) | 153 (76.5) |
| Admission | 366 (81.3) | 171 (85.5) |
| 30-day mortality, n (%) | 51 (11.3) | 30 (15.0) |

Values are *n* (%) unless stated otherwise.

^a^In total, 450 patients were included in the derivation cohort during the prospective study period in Zuyderland MC (included patients). The non-included patients represent a sample of 200 patients who were not included during the prospective study period for reasons other than refusal.

^b^There were no significant differences in baseline characteristics between patient who were included and patients who were not included.

*CCI* Charlson comorbidy Index score; *IQR* interquartile range

| **Table S3.** *Results of follow up echocardiogram in patients with MACE* | |
| --- | --- |
| **Echocardiographic finding** | ***n*=41** |
| **New findings on echocardiogram** |  |
| Further or new decrease in left ventricle function | 5 |
| Further or new decrease in left ventricle function in combination with heart valve disease | 2 |
| Increase in pulmonary hypertension | 2 |
| Acute septum rupture | 1 |
| Pericardial effusion | 1 |
| **Stable heart function on echocardiogram** |  |
| Stable valve disease | 1 |
| Stable impaired left ventricle function in combination with heart valve disease | 2 |
| **No earlier echocardiogram** |  |
| Impaired left ventricle function | 5 |
| Heart valve disease | 7 |
| Impaired left ventricle function in combination with heart valve disease | 1 |
| Pulmonary hypertension | 1 |
| **Normal echocardiogram** | 13 |
|  |  |
| **Median Time to echocardiogram, days (IQR)** | **76 (17 - 288)** |

**References**

1. Tan SY, Sungar GW, Myers J, et al. A simplified clinical electrocardiogram score for the prediction of cardiovascular mortality. Clin Cardiol. 2009;32(2):82-6. doi: 10.1002/clc.20288.
